# The Role of the CuCl Active Complex in the Stereoselectivity of the Salt-Induced Peptide Formation Reaction: Insights from Density Functional Theory Calculations

**DOI:** 10.3390/life13091796

**Published:** 2023-08-23

**Authors:** Allison C. Fox, Jason D. Boettger, Eve L. Berger, Aaron S. Burton

**Affiliations:** 1 NASA Postdoctoral Program, NASA Johnson Space Center, Houston, TX 77058, USA; 2 Department of Earth, Environmental and Resource Sciences, University of Texas at El Paso, El Paso, TX 79968, USA; jasondboettger@gmail.com; 3 Astromaterials Research and Exploration Science Division, NASA Johnson Space Center, Houston, TX 77058, USA

**Keywords:** amino acid, biohomochirality, polymerization, salt-induced peptide formation, stereoselective

## Abstract

The salt-induced peptide formation (SIPF) reaction is a prebiotically plausible mechanism for the spontaneous polymerization of amino acids into peptides on early Earth. Experimental investigations of the SIPF reaction have found that in certain conditions, the l enantiomer is more reactive than the d enantiomer, indicating its potential role in the rise of biohomochirality. Previous work hypothesized that the distortion of the CuCl active complex toward a tetrahedral-like structure increases the central chirality on the Cu ion, which amplifies the inherent parity-violating energy differences between l- and d-amino acid enantiomers, leading to stereoselectivity. Computational evaluations of this theory have been limited to the protonated–neutral l + l forms of the CuCl active complex. Here, density functional theory methods were used to compare the energies and geometries of the homochiral (l + l and d + d) and heterochiral (l + d) CuCl–amino acid complexes for both the positive–neutral and neutral–neutral forms for alanine, valine, and proline. Significant energy differences were not observed between different chiral active complexes (i.e., d + d, l + l vs. l + d), and the distortions of active complexes between stereoselective systems and non-selective systems were not consistent, indicating that the geometry of the active complex is not the primary driver of the observed stereoselectivity of the SIPF reaction.

## 1. Introduction

Given the role of peptides, and eventually proteins, in performing catalytic and structural functions, the formation of amino acid polymers from their monomers was a critical step for the evolution of life on Earth. Peptides are formed via condensation polymerization, where the amino group of one amino acid reacts with the carboxylic acid group of a second amino acid, forming an amide bond and releasing a water molecule in the process. Because water is a by-product of polymerization, uncatalyzed peptide bond formation is thermodynamically unfavorable in the aqueous environments predicted to have existed on prebiotic Earth [1]. This is further compounded by the fact that the reverse reaction of condensation is hydrolysis, which is favored when water activity is high [2]. Several mechanisms have been proposed to provide a prebiotically plausible route to peptide formation under early Earth conditions, including mineral catalysts [3,4,5,6], condensation reagents [7,8], metal ions [9] or environmentally mediated dehydration such as wet–dry cycling [10,11]. 

**Figure 1:**
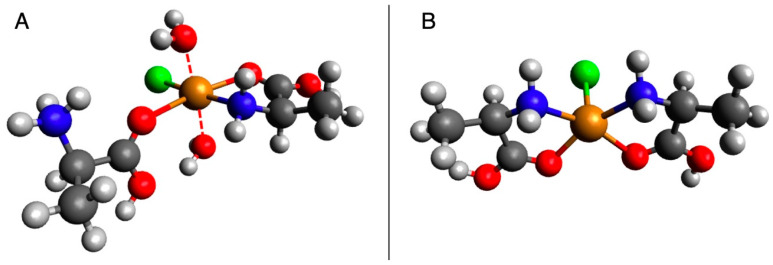
(**A**) The proposed active complex proposed by Rode et al. (1990) for the SIPF reaction for a protonated and neutral alanine molecule [18]. (**B**) Another plausible SIPF active complex between two neutral alanine molecules in a cis configuration as proposed by Rimola et al. (2007) [20]. Here, *cis* refers to the relative position of the N (or equivalently O) atoms on the same side of the plane containing the Cu atom. In this figure, red denotes oxygen, blue denotes nitrogen, gray denotes carbon, light gray denotes hydrogen, orange denotes copper, and green denotes chlorine.

In scenarios invoking dehydrating conditions, salts reduce water activity and facilitate polymerization, thus reducing the thermodynamic barrier to polymerization in aqueous environments. One scenario that has been investigated extensively is the salt-induced peptide formation (SIPF) reaction [12,13,14,15,16,17,18,19]. In laboratory experiments, the SIPF reaction medium is a salt solution containing monovalent and divalent metal ions (most commonly, NaCl and CuCl_2_, respectively). The Na, Cl, and Cu ions aid in reducing water activity, thus helping to drive the reaction toward peptide formation, while the divalent Cu additionally lowers the activation energy for peptide formation. The Cu ion readily forms complexes with multiple α-amino acids, bringing them into close proximity and activating reactive moieties. The originally proposed reactive species consisted of one neutral amino acid chelated with the copper–chloride complex, while a protonated second amino acid binds end-on via its carboxyl group, and two water molecules remain bound to the complex, one above, one below (hereafter referred to as the protonated–neutral complex; see Figure 1A) [17]. Subsequent work has also identified a bi-bidentally bound CuCl–amino acid complex between two neutral amino acids species as a potential reactive species (hereafter referred to as the neutral–neutral species, Figure 1B) [20]. The formation of these complexes mediates amide bond formation between the amino acids via a nucleophilic attack by the amine group of the bidentate amino acid (amino acid 2) on the carboxyl carbon of amino acid 1. The complexation of the Cu (II) is weaker than the newly formed amide bond, and the Cu (II) ion is released back into solution. Further elongation of the peptide occurs via the same mechanism, with the nascent peptide binding end-on via its carboxyl group and a free amino acid chelating the copper–chloride complex; one amino acid can be added per polymerization cycle.

The requirements to facilitate the SIPF reaction are feasible in an early Earth environment:

(1) *Amino Acids.* Amino acids either formed in situ in prebiotic environments [21,22,23] or delivered via meteorites [24,25,26].
(2) *Divalent Cations.* Although other metal ions can catalyze the reaction (e.g., Cr (III), Al (III), Mg (II)), divalent copper confers the best reactivity [17,18]. Cu-bearing deposits (known as ‘greenstones’) found in Precambrian rock formations indicate that sufficient Cu(II) was present to facilitate the SIPF reaction in prebiotic environments [27].
(3) *Chloride Salts.* NaCl was likely present in prebiotic environments, either leached from the Earth’s crust, which contains 130–1910 ppm of chlorine and 2.0–2.9 wt% sodium [28], into freshwater sources, or were sourced from water entering the reaction environments from oceans or lagoons.
(4) *Temperature.* Experimental work has found the highest peptide yields were in SIPF reactions performed at temperatures between 60 and 90 °C, which is not unreasonable for a recently condensed primordial ocean or surface temperatures of the early Earth [9].
(5) *pH.* Optimal pH conditions for the SIPF reaction should be below 3 to prevent Cu-catalyzed peptide hydrolysis and above 2 to prevent proton-catalyzed peptide hydrolysis. Acidic environments on prebiotic Earth, such as acidic hot springs etc., could provide suitable pH conditions.

An intriguing outcome of previous studies of the SIPF reaction is the reported enhanced reactivity of l-amino acid enantiomers over their d-amino acid counterparts. For valine, for example, Plankensteiner and co-workers (2005a) reported divaline yields that were as much as 20- to 380-fold for ll peptides than dd peptides formed via the SIPF reaction [29]. Separately, Plankensteiner and co-workers (2005b) measured the chiral selectivity of peptide formation of the SIPF reaction with several individual amino acids [15]. This work revealed maximum selectivities for ll over dd peptides of the following amounts depending on the reaction conditions (peptide, (ratio of ll/dd)): divaline (408.33); diproline (1.69); ditryptophan (2.54); dilysine (1.32); and diserine (1.38). However, these reactions were performed as independent enantiopure reactions that only contained all l- or all d-enantiomers. This can be problematic because different enantiopure enantiomers of a given amino acid often come from different sources, such as extraction from biological materials for the naturally occurring l-enantiomers of amino acids versus the chemical synthesis of the d-enantiomers. This can lead to the presence of different impurities, which could have impacted the reactions discussed above. For example, previous work found that l-tyrosine was more soluble than d-tyrosine, which was initially attributed to parity violating energy differences between the enantiomers [30]. However, when these experiments were repeated by Lahav and co-workers (2006) using amino acids sourced from several different companies, the results were mixed, with some experiments showing l-tyrosine as more soluble than, equally soluble as, or less soluble than d-tyrosine [31]. In addition, assays of the samples revealed that they each contained different species and abundances of contaminants, strongly implying that these impurities likely played a significant role in the observed apparent differences in solubility. Thus, it remains a possibility that the chiral selectivity preferences for ll enantiomers observed in the SIPF reaction reported to date could have been similarly affected by differences in impurities between the two enantiomers. 

Setting aside potential issues with enantiopure reactions, Plankensteiner et al. (2005b) postulated that the stereoselectivity of the SIPF reaction they observed could be the result of differences in geometry between different complexes that amplified the polarity-violating energetic differences between the two enantiomers [15]. In order to evaluate the plausibility of this hypothesis, we use density functional theory calculations to compare the stability and structure of heterochiral and homochiral complexes of the individual amino acids alanine, valine and proline, in scenarios containing d + d, l + d, and l + l enantiomer pairings for both the protonated–neutral and neutral–neutral complexes. These amino acids were chosen to explore the effects of smaller (alanine) and bulkier (valine) side-chains as well as the effects of having a secondary amine (proline) versus the primary amines alanine and valine. In addition, valine was also chosen because it was reported to show the largest stereoselectivity in the SIPF reaction with over a 400-fold increase in reactivity between l + l and d + d SIPF reactions. 

## 2. Materials and Methods

### 2.1. Computational Details

Previous works have found that functionals with a larger percentage of exact exchange (50%), including the BH and HLYP method, compare better to the highly correlated CCSD(T) method for open-shell systems than the more commonly used B3LYP method [32,33]. Subsequent studies comparing these methods for Cu–amino acid complexes have found BH and HLYP relative Gibbs energies to be larger by 10–30 kJ mol^−1^, and they attribute the differences to the shortcomings of the B3LYP method to describe the delocalized nature of the Cu^2+^ complexes [20,34,35].

Therefore, all geometries of the CuCl—(alanine)_2_, CuCl—(valine)_2_, and CuCl—(proline)_2_ complexes were completely optimized using the density functional theory method BH and HLYP/6-311++G(d,p) [36] employed by the Gaussian 09 program, revision D.01 [37]. The free energies for each model were calculated in the Gaussian 09 program and represent the sum of the electronic and thermal free energies [38]. For each neutral–neutral complex, two homochiral (l + l and d + d) and the heterochiral (l + d) complexes in the *cis* and *trans* formation were optimized for a total of six models per Cu–amino acid pairing. In this context, *cis* and *trans* refer to the relative orientation of the chelating N and O atoms of the amino acids, with *trans* models involving roughly linear O-Cu-O and/or N-Cu-N axes (i.e., O are *trans* from one another relative to the central Cu, as are N), and *cis* models involving roughly linear O-Cu-N axes and N atoms (or equivalently O atoms) that are on the same side (*cis*) of the Cu atom (e.g., [39]). For the protonated–neutral complexes, both homochiral and the heterochiral forms were modeled for a total of three models per Cu–amino acid pairing. The influence of solvation for each structure was modeled using the polarizable continuum model [40]. The models reached a local energy minimum, as indicated by a lack of imaginary frequencies in the harmonic results. Avogadro was used for the preparation, manipulation, and visualization of the structures [41].

Although previous work has calculated molecular parity violation (PV) for chiral metal—acetylacetonate complexes using relativistic DFT calculations [42], the DFT methods employed here are not sufficient to measure energy differences driven by PV effects between l+ l and d + d complexes. However, energy differences between homochiral and heterochiral complexes are potentially observable via these methods if driven by something other than PV effects, such as steric effects. Furthermore, these methods will provide optimized geometries of the CuCl–amino acid complexes comparable to previous work [13] to evaluate the role of geometry in potentially amplifying PV effects. 

### 2.2. Calculations

To assess the degree of chirality for each optimized CuCl–amino acid complex, the continuous chirality measure (CCM) was used. The CCM is calculated by finding the minimum distance that the vertices of a selected molecule need to shift in order to attain an achiral symmetry. For a given chiral molecule, Q, with N vertices whose 3N Cartesian coordinates, q_k_, are arranged in N vectors, q_i_, there exists a nearest achiral molecule, G, whose Cartesian coordinates, p_k_, are organized in N vectors p_i_. The symmetry measure of Q with respect to G is defined as:

$$S_{Q}\left(G\right)=\min\left[\frac{\sum_{i=1}^{N}{\left|\vec{q_{l}}-\vec{p_{l}}\right|}^{2}}{\sum_{i=1}^{N}{\left|\vec{q_{l}}-\vec{q_{0}}\right|}^{2}}\right]\times 100$$

In this equation, *q*_0_ is the position vector of the geometric center of *Q*, and the denominator is the mean square size normalization factor. A zero value for a *S*(*G*) indicates the perfect symmetry or achirality of the molecule [43]. All CCM calculations were performed using the CoSyM calculator (http://csm.ouproj.org.il, accessed 1 January 2023) with the maximal degree, *S_n_* = 8 [44].

Torsion angles are calculated to assess the degree of coplanarity of Cu model complexes. Angles were computed between the plane defined by the Cu–bidentate amino acid ligand (defined by the positions of the ligating O and N atoms) and the bond between the Cu and monodentate ligand. The angle is computed as the complement of the angle between the Cu–monodentate vector and the vector perpendicular to the Cu–bidentate plane (computed via the Cu-O and Cu-N cross-product). Monodentate ligands coplanar with the Cu-bidentate plane thus give an angle of 0 degrees; deviations from this value give the degree of non-coplanarity. 

## 3. Results

### 3.1. Gibbs Free Energy

**Figure 2:**
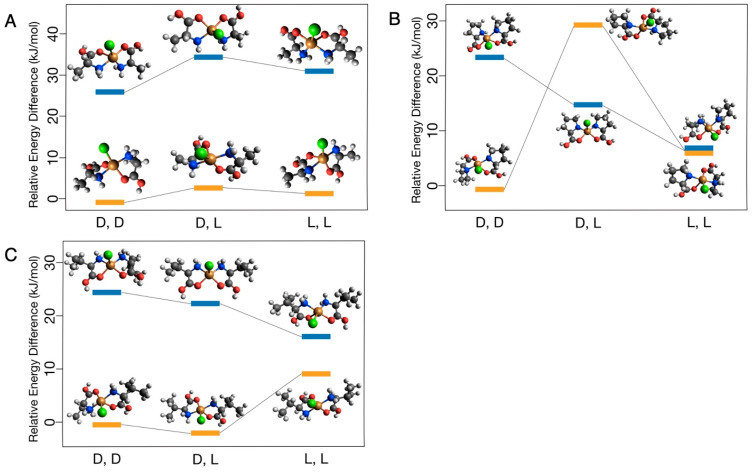
Calculated free energy differences between the neutral–neutral CuCl complexes of (**A**) alanine, (**B**) proline, and (**C**) valine in the cis (blue) and trans (yellow) configuration.

**Figure 3:**
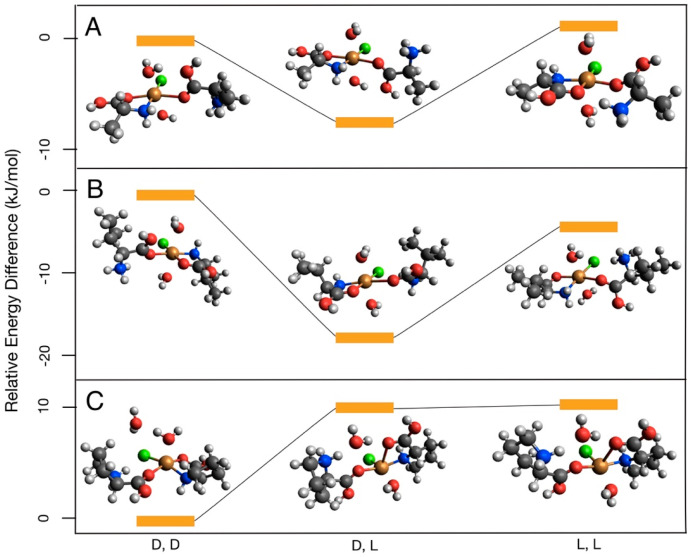
Calculated free energy differences between the protonated–neutral CuCl complexes of (**A**) alanine, (**B**) valine, and (**C**) proline.

The Gibbs free energies relative to the appropriate dd complex are reported in Figure 2 and Figure 3, with corresponding values listed in Table 1 and Table 2. For the neutral–neutral amino acid CuCl complexes, values vary from ~1 to 30 kJ/mol with no consistent pattern between energy differences and chirality. Significant energy differences were observed when comparing *trans* and *cis* isomers, with complexes in the *trans* configuration having generally lower Gibbs free energy values beyond the expected DFT chemical accuracy of ~4 kJ/mol (Figure 2). Similarly, the protonated–neutral amino acid CuCl complexes vary from <1 to 19 kJ/mol with no consistent trends observed (Figure 3). Theoretically, energy differences between otherwise symmetrical homochiral complexes (ll and dd) would be driven by relatively small parity violating energy differences (PVEDs) [45]. Inherent PVEDs are very small, approximately 10^−13^ kJ/mol for Cu and 10^−16^ kJ/mol for alanine. Although these differences are potentially amplified by the formation of the CuCl–amino acid complex, energy differences are still expected to be <1 kJ/mol between homochiral complexes [16]. The much larger observed energy differences in both model types are more likely the consequence of rotations within the model during early stages of the geometry optimization, which locate different local minimum energy structures as a result. To confirm this, a subset of models was run from varied starting geometries. The standard deviations of these runs varied from <1 to 19 kJ/mol, indicating that local minimum energy structures can differ by approximately this amount of energy due solely to minor variations in the starting atom position, and that the observed energy differences are likely driven by rotations within the model and not differences in the inherent stability of the complex. Some examples of the geometric differences which can contribute to differences in energy of the homochiral complexes can be seen in Figure 2 and Figure 3; e.g., the orientation of *cis* proline carboxyl -OH groups (Figure 2B), the orientation of H_2_O relative to one another and to other ligands (Figure 3B), and the final position of the sixth ligand (Figure 3C), which is thought to interact weakly with Cu, resulting in five-fold coordination [46].

### 3.2. Torsion Angles and Geometry

Two measurements were used to compare the geometries of amino acid CuCl complexes: the distance between O and N atoms of opposing amino acids and the distortion of the equatorial plane represented by the torsion angle. In the neutral–neutral complexes, the O-N distances were minimized in the *trans* configuration relative to the *cis* configuration. Additionally, within the *cis* isomers, heterochiral complexes had larger O-N distances compared to homochiral complexes, but this trend was not observed in the *trans* isomers (Table 3). For the protonated–neutral complexes, O-N distances did not follow consistent trends with chirality (Table 4).

**Figure 4:**
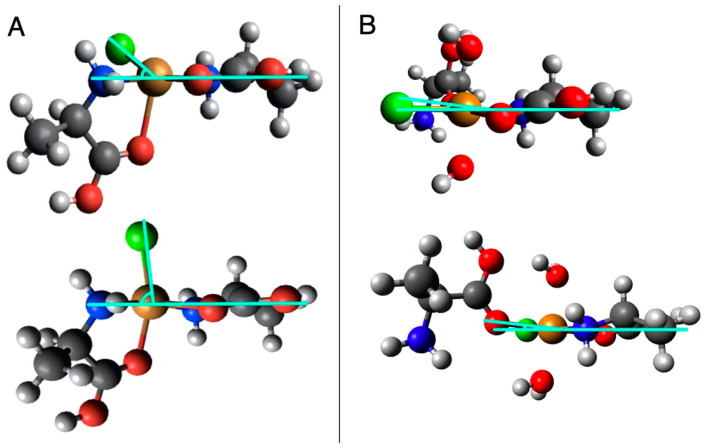
Torsion angles (indicated in cyan) between the plane formed by the Cu and bidentally bound amino acid and (**A**) each of the other bidentally bound amino acids in the neutral–neutral complexes and (**B**) the Cl ligand (top) and end-on amino acid (bottom) in the protonated–neutral complexes.

Torsion angles were used to describe the distortion of the equatorial plane toward a tetrahedral structure. For the neutral–neutral complexes, two planes are formed between the chloride ligand and the copper ion and each of the bidentally bound amino acids (Figure 4A). Torsion angles for these complexes did not show consistent trends with chirality, amino acid type, or configuration (Table 5). In the protonated–neutral complexes, torsion angles were significantly lower than the neutral–neutral Cu–amino acid complexes and similarly did not show trends with chirality (Table 6). Torsion angles in these models are formed between either the ligand or the end-on bound amino acid and the plane formed by the copper ion the bidentally bound amino acid (Figure 4B). The largest torsion angles were observed in the proline models, which is inconsistent with previous computational work [13].

### 3.3. Continuous Chirality Measure

**Figure 5:**
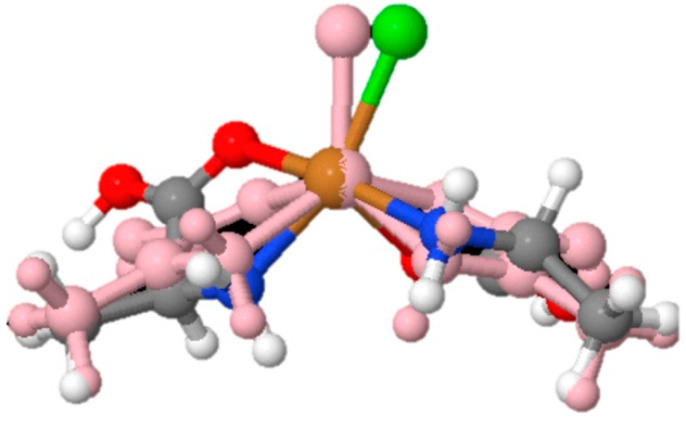
The geometry of the achiral structure (pink) compared to the chiral structure for the (d,d) ala2-CuCl complex.

The continuous chirality measure (CCM) provides a metric to quantify the degree of chirality in a molecule by measuring the deviation of the structure of the molecule from an achiral point group [43]. Figure 5 shows the geometry of the achiral structure compared to the chiral structure that is the basis of the CCM for the (d + d) ala_2_-CuCl complex. For the neutral–neutral complexes, CCM values range from ~0 to 14 (Table 7), with higher values correlated with larger deviations from the achiral structure. Generally, *trans* isomers have a higher CCM value than *cis* isomers. Within the *cis* isomer group, homochiral complexes are more chiral than heterochiral complexes. In the *trans* configuration, alanine complexes have the highest CCM values, which is followed by proline and then valine complexes. The opposite trend is observed in the *cis* amino acid complexes. For the protonated–neutral models, CCM values varied from ~3 to 7 (Table 8). In alanine and valine complexes, the highest CCM values were observed in the homochiral complexes, while the (d + d) proline_2_-CuCl had the highest CCM among proline complexes. Overall, the highest CCM values were observed in valine followed by alanine and then proline.

## 4. Discussion

### 4.1. Differences in the Free Energy of CuCl–Amino Acid Complexes

Experimental investigations of the SIPF reaction have reported approximately 10–20% higher yields for the l + l dipeptide over the d + d peptide for several amino acids including alanine, tryptophan, lysine, arginine, and serine and as much as 400-fold higher for valine [12,14,15,16,29]. The apparent preference for the l + l peptide has been attributed to very small parity violating energy differences (PVEDs) inherent to l and d enantiomers that are amplified by the CuCl–amino acid complex geometry [13]. However, these experiments were performed on enantiopure reactions containing only l- or only d-enantiomers, where each enantiomer is typically obtained from a different source (e.g., biology versus chemically synthesized), leading to potential differences in purity and contaminant composition. This was an issue for a study that reported differences in the solubility of d- and l-tyrosine, which were later found to be the result of different contaminants in the d- and l-tyrosine samples [30,31]. 

In this work, we compared the free energies of l + l and d + d vs. l + d CuCl–amino acid complexes to evaluate how chirality affected the stability of the complexes. Although energy differences due to PVEDs between enantiomers can be amplified by certain chemical processes, experimental work comparing circular dichroism spectra of Cu-l-alanine, Cu-d-alanine, and Cu-racemic mixture systems show only slight variations, indicating that even amplified energy differences are likely very small [16]. Even in experiments with ~10–20% higher dipeptide yields for l-amino acids over d -amino acids, the calculated difference in the free energies between the l-amino acid reaction and d -amino acid reaction are still less than 1 kJ/mol [12,14,16]. As a result, the small energy differences due to PVEDs for CuCl–amino acid complexes are not measurable with the chosen computational tools. We attribute the observed energy differences between l + l and d + d complexes to rotations of various functional groups within a model. These energy differences do not reflect differences in the stability of the l + l and d + d complexes. We found that rotations of various functional groups within a model can account for differences up to 19 kJ/mol. The high molecular degrees of freedom in the large CuCl–amino acid systems complicate our evaluation of energy differences between the homochiral vs. heterochiral complexes that could be driven by steric effects. We did not observe consistent trends for homochiral vs. heterochiral complexes (Figure 2 and Figure 3), suggesting that either these energy differences do not exist or they are masked by energy differences caused by rotations of various functional groups within a model.

Among the neutral–neutral amino acid CuCl complexes, energy differences larger than those expected from molecular rotations were observed between the *cis* and *trans* isomers (Figure 2). For the alanine and valine complexes, the *trans* isomers had lower free energies than their corresponding *cis* isomers, which is consistent with previous modeling work that found *trans* Cu-gly_2_ complexes were more stable than corresponding *cis* isomers [20]. In experimental work, the equilibrium between *cis* and *trans* isomers for Cu–amino acids complexes is controlled by the pH and polarity of the solution. Generally, the *trans* isomers are preferred at lower pHs and in less polar solutions [47,48]. Unlike the alanine and valine complexes, the *trans* isomers of the proline complexes were not energetically favored compared to the *cis* isomers (Figure 2). Bukharov et al. (2014) did not see significant differences in the relative abundance of *cis* and *trans* isomers of Cu–proline complexes compared to Cu–alanine and Cu–valine complexes, indicating the observed differences are likely a consequence of rotations within the proline models rather than stability differences in *cis* and *trans* isomers.

### 4.2. Differences in the Geometry of CuCl–Amino Acid Complexes

#### 4.2.1. Geometry of Protonated–Neutral Proline Complexes

Among the protonated–neutral complexes, geometry optimization always favored the de-complexation of one H_2_O molecule and the formation of a distorted square pyramidal structure with only five explicit ligands. Subsequent attempts at re-optimization could not locate a local minimum in which all six valence sites were occupied by explicit ligands, implying that in these cases, coordination by H_2_O as a sixth ligand is energetically unfavorable, and loss of the sixth ligand may be barrierless. Distortions are thought to occur in some Cu complexes in aqueous solution and involving ligands with carboxyl and amino groups resulting in the occupation of only five valence sites. Combined neutron diffraction measurements and molecular dynamics models favor the coordination of Cu^2+^ by five H_2_O molecules in solution [49], specifically with four equidistant H_2_O ligands implying a square planar geometry [50]. The coordination of Cu^2+^ by five ligands in solution is also supported by X-ray absorption spectroscopy results [51,52]. Extended X-ray absorption fine structure measurements of Cu^2+^ coordination in biofilms indicate that most of the Cu is coordinated by 5.1 ± 0.3 O or N atoms [53]. Complexes between Cu^2+^ and EDTA at the TiO_2_ surface investigated with electron paramagnetic resonance spectroscopy appear to display coordination by only five groups: two amines and two carboxylates from EDTA, and a single H_2_O molecule [46]. Five-fold coordination by EDTA has also been invoked to explain X-ray photoelectron spectroscopy observations of the binding energy of the Cu 2p_3/2_ electrons [54]. Structures with both five and six coordinating groups are thought to coexist in solution [55], leading to the existence of some models favoring five ligands, while some favoring six is not surprising.

#### 4.2.2. Torsion Angles for CuCl–Amino Acid Complexes 

Previous work attributed the degree of stereoselectivity observed in the SIPF reaction to the distortion of the CuCl–amino acid reactive species. In this hypothesis, the active complex twists toward a tetrahedron-like structure, which induces a central chirality at the Cu ion. The imposed chirality coupled with the inherent chirality of Cu, which is significantly stronger due to its higher atomic number, and it allows the CuCl complex to act as a chemical amplifier for the small PVEDs inherent to l and d enantiomers. As a result, CuCl complexes that are more distorted will lead to larger parity-violating energy differences between the l-amino acid complex and its d-counterpart, increasing the stereoselectivity of the SIPF reaction [13,16]. 

The torsion angle, or the angle formed between the monodentally bound ligand (i.e., the Cl atom or amino acid) and the plane formed by the copper ion and bidentally bound amino acid, can be used to quantify this distortion of a complex (Figure 4). Previous work found that the torsion angles measured from DFT-calculated structures of the l + l-CuCl-ala_2_ complexes, which show a preference for the l-form in experimental work, were slightly larger, ~5°, than torsion angles observed in the l + l-CuCl-pro_2_ complexes, which show no preference [13], indicating that the more distorted alanine SIPF complex leads to its observed stereoselectivity. We similarly calculated torsion angles for the end-on amino acid in the l + l alanine, valine, and proline complexes (Table 6) but found the difference in torsion angle between alanine and proline CuCl complexes was just one degree. Furthermore, when torsion angles are compared for the d + d and l + d complexes, there is not a consistent pattern, with proline having the largest torsion angle among the d + d complexes and alanine having the largest torsion angle among the l + d complexes. The differences between our work and Fitz et al. (2007) are likely due to small differences in the geometry of the complexes, which were calculated with different basis sets. 

Direct comparisons between the torsion angles for the protonated–neutral complexes and the neutral–neutral complexes are difficult given the double bidentate structure of the neutral–neutral complexes. However, it is clear from both the torsion angles and visual inspection of the models that the neutral–neutral complexes are significantly more ‘distorted’ than the protonated–neutral complexes. Among the neutral–neutral complexes, there are not consistent distortion trends; i.e., for some chiralities, proline is more distorted than alanine or valine and vice versa. Our models for both the protonated–neutral and neutral–neutral CuCl–amino acid complexes do not indicate that the distortion of the complex would be a predictor of stereoselectivity in experimental results. 

#### 4.2.3. Continuous Chirality Measure (CCM) for CuCl–Amino Acid Complexes

To better compare the distortion of the protonated–neutral and neutral–neutral CuCl–amino acid complexes, the continuous chirality measure was used to measure each complex’s deviation from an achiral structure. If the hypothesis presented in Fitz et al. (2007) is correct, we would expect to see higher CCM values for alanine and valine complexes, which show an l-enantiomer preference in experimental work, than proline complexes, which do not. Similar to the torsion angle results, we did not observe consistent trends for CCM values based on amino acid type (i.e., alanine vs. proline vs. valine). Among the protonated–neutral complexes, there are no trends between amino acid type, but generally, l + l complexes have the lowest CCM value (Table 8). The neutral–neutral complexes also did not show trends with amino acid type but reflected the torsion angle results, with the *trans* isomers having higher CCM values than their corresponding *cis* isomers. These results are not consistent with the hypothesis of Fitz et al. (2007), but they do indicate that *trans* isomers are significantly more distorted than *cis* isomers. This distortion decreases the distance between the O and N atoms of opposing amino acids that will eventually form a peptide in the *trans* isomers (Table 3), which may lower the activation energy of polymerization.

## 5. Conclusions

The present calculations suggest that energy differences between homochiral and heterochiral Cu–amino acid complexes, if present, are small enough to be obscured by rotations of functional groups within the models. However, for the neutral–neutral Cu–amino acid complexes, energy differences between trans and cis isomers were not masked by molecular rotations, with trans isomers generally being energetically preferred. This work suggests that smaller, less complex models should be explored to compare the stability of heterochiral and homochiral SIPF complexes in tandem with laboratory experiments. Comparisons of the structures of the lowest energy, protonated–neutral Cu–amino acid complexes were not consistent with previous work that found more ‘distorted’ complexes in amino acids that reportedly showed increased stereoselectivity in experimental investigations. Consistent trends for both torsion angle and CCM among amino acid types were not observed, indicating that distortion of the Cu–amino acid complex is not driving the reported stereoselectivity of the SIPF reaction. Among the neutral–neutral Cu–amino acid complexes, the trans isomers were found to be more distorted and have smaller O-N distances compared to cis isomers, suggesting that trans isomers may better facilitate polymerization. Based on these results, future experimental work should prioritize the performance of: (1) SIPF experiments using racemic mixtures of amino acids to confirm that the stereoselectivity of the reaction is not due to different impurities in commercial l and d amino acid sources; (2) SIPF experiments at varying pH values to determine if amino acid mixtures with higher proportions of neutral amino acids affect polymerization rates, and (3) SIPF experiments in solutions of varying polarity that would influence the proportions of cis vs. trans isomers.

## Figures and Tables

**Table 1:** Free energy differences from the d + d models for the neutral–neutral complexes at 25 °C.

Amino Acid	Enantiomers	Configuration	Energy Difference from d + d Model (kJ/mol)
Alanine	d + l	*Trans*	3.47
Alanine	l + l	*Trans*	2.11
Alanine	d + l	*Cis*	5.92
Alanine	l + l	*Cis*	3.51
Valine	d + l	*Trans*	−1.77
Valine	l + l	*Trans*	8.68
Valine	d + l	*Cis*	−1.61
Valine	l + l	*Cis*	−7.52
Proline	d + l	*Trans*	29.37
Proline	l + l	*Trans*	5.35
Proline	d + l	*Cis*	−8.26
Proline	l + l	*Cis*	−16.45

**Table 2:** Free energy differences from the d + d models for the protonated–neutral complexes at 25 °C.

Amino Acid	Enantiomers	Energy Difference from d + d Model (kJ/mol)
Alanine	d + l	−7.73
Alanine	l + l	0.95
Valine	d + l	−17.29
Valine	l + l	−6.17
Proline	d + l	10.27
Proline	l + l	10.69

**Table 3:** Distance between O and N atoms in opposing amino acids for the neutral–neutral amino acid CuCl complexes.

Amino Acid	Enantiomers	Configuration	O-N Distance (Å)	O-N Distance (Å)
Alanine	d + d	*Trans*	2.906	3.272
Alanine	d + l	*Trans*	2.916	3.181
Alanine	l + l	*Trans*	2.917	3.226
Alanine	d + d	*Cis*	3.26	4.041
Alanine	d + l	*Cis*	4.001	4.007
Alanine	l + l	*Cis*	3.259	4.043
Valine	d + d	*Trans*	2.905	3.288
Valine	d + l	*Trans*	2.908	3.340
Valine	l + l	*Trans*	2.965	3.163
Valine	d + d	*Cis*	4.031	4.022
Valine	d + l	*Cis*	4.017	4.022
Valine	l + l	*Cis*	3.196	4.071
Proline	d + d	*Trans*	2.839	3.473
Proline	d + l	*Trans*	2.904	3.233
Proline	l + l	*Trans*	2.844	3.426
Proline	d + d	*Cis*	3.257	4.033
Proline	d + l	*Cis*	3.995	4.024
Proline	l + l	*Cis*	3.567	4.039

**Table 4:** Distance between O and N atoms in opposing amino acids for the protonated–neutral amino acid CuCl complexes.

**Amino Acid**	**Enantiomers**	**O-N Distance (Å)**
Alanine	d + d	3.148
Alanine	d + l	3.140
Alanine	l + l	4.066
Valine	d + d	4.115
Valine	d + l	4.759
Valine	l + l	3.079
Proline	d + d	2.876
Proline	d + l	3.963
Proline	l + l	3.975

**Table 5:** Torsion angles formed between the Cl ligand and the plane formed by the copper ion and each of the bidentally bound amino acids (represented at bidentate planes 1 and 2) in the neutral–neutral complexes.

Mino Acid	Enantiomers	Configuration	Bidentate Plane 1	Bidentate Plane 2
Alanine	d + d	*Trans*	21.75	71.54
Alanine	d + l	*Trans*	25.65	65.70
Alanine	l + l	*Trans*	25.14	65.14
Alanine	d + d	*Cis*	20.77	75.12
Alanine	d + l	*Cis*	77.83	76.90
Alanine	l + l	*Cis*	20.96	75.07
Valine	d + d	*Trans*	20.94	74.49
Valine	d + l	*Trans*	20.76	74.86
Valine	l + l	*Trans*	31.20	57.93
Valine	d + d	*Cis*	77.01	78.28
Valine	d + l	*Cis*	77.46	77.86
Valine	l + l	*Cis*	16.94	75.78
Proline	d + d	*Trans*	17.34	75.10
Proline	d + l	*Trans*	22.98	66.97
Proline	l + l	*Trans*	20.91	75.60
Proline	d + d	*Cis*	30.84	58.40
Proline	d + l	*Cis*	73.27	75.21
Proline	l + l	*Cis*	20.90	75.60

**Table 6:** Torsion angles formed between either the Cl ligand or the end-on bound amino acid and the plane formed by the copper ion the bidentally bound amino acid in the protonated–neutral complexes.

Amino Acid	Enantiomers	Cl Ligand	End-On Amino Acid Ligand
Alanine	d + d	0.67	1.71
Alanine	d + l	0.45	2.7
Alanine	l + l	1.45	2.24
Valine	d + d	0.09	0.74
Valine	d + l	0.73	0.98
Valine	l + l	1.18	5.74
Proline	d + d	17.58	15.02
Proline	d + l	65.81	0.44
Proline	l + l	68.28	1.24

**Table 7:** Continuous chirality measure for the neutral–neutral monochlorocuprate complexes.

Amino Acid	Enantiomers	Configuration	CCM (Ln = 8)
Alanine	d + d	*Trans*	13.7745
Alanine	d + l	*Trans*	11.4749
Alanine	l + l	*Trans*	9.3129
Alanine	d + d	*Cis*	4.5793
Alanine	d + l	*Cis*	0.3502
Alanine	l + l	*Cis*	4.3117
Valine	d + d	*Trans*	9.2988
Valine	d + l	*Trans*	5.5452
Valine	l + l	*Trans*	6.1477
Valine	d + d	*Cis*	6.7445
Valine	d + l	*Cis*	0.0114
Valine	l + l	*Cis*	6.0647
Proline	d + d	*Trans*	8.6882
Proline	d + l	*Trans*	10.6915
Proline	l + l	*Trans*	9.3419
Proline	d + d	*Cis*	8.1805
Proline	d + l	*Cis*	1.7808
Proline	l + l	*Cis*	9.3426

**Table 8:** Continuous chirality measure for the hydrated protonated–neutral monochlorocuprate complexes.

Amino Acid	Enantiomers	CCM (Ln = 8)
Alanine	d + d	5.2802
Alanine	d + l	5.8559
Alanine	l + l	3.2276
Valine	d + d	6.7988
Valine	d + l	7.2371
Valine	l + l	5.6539
Proline	d + d	5.6731
Proline	d + l	4.2988
Proline	l + l	3.4478

## Data Availability

The data presented in this study are openly available in the Harvard Dataverse at https://doi.org/10.7910/DVN/AW36KS (Fox, 2023; accessed 1 March 2023).
